# Sir Benj. Brodie

**Published:** 1860-10

**Authors:** 


*Sir Benj. Brodie* is still suffering from impaired sight. The operation performed upon him of iridectomy has not been successful. The hope is, that it may prove a cataract which can be extracted. The loss to science of this able man would prove a world-wide calamity. B.

